# Molecular Characterization of Gram-Negative Bacteria: Antimicrobial Resistance, Virulence and Epidemiology

**DOI:** 10.3390/antibiotics13050402

**Published:** 2024-04-28

**Authors:** Theodoros Karampatakis

**Affiliations:** Microbiology Department, Papanikolaou General Hospital, 57010 Thessaloniki, Greece; tkarampatakis@yahoo.com

Multidrug-resistant (MDR), extensively drug-resistant (XDR) and pan-drug-resistant (PDR) Gram-negative bacteria constitute a huge public health problem. According to the European Centre for Disease Prevention and Control (ECDC), MDR is defined as ‘acquired nonsusceptibility to at least one agent in three or more antimicrobial categories’ and extensively drug-resistant is defined as ‘nonsusceptibility to at least one agent in all but two or fewer antimicrobial categories (i.e., bacterial isolates remain susceptible to only one or two categories)’. PDR is defined as ‘nonsusceptibility to all agents in all antimicrobial categories’ [1]. Among them, MDR Gram-negative bacteria and especially *Klebsiella pneumoniae*, *Pseudomonas aeruginosa*, and *Acinetobacter baumannii* are the main bacteria leading to increased rates of antimicrobial resistance [2]. Infections caused by these MDR bacteria adversely affect the outcome of patients’ hospitalization and increase mortality rates [3]. In addition, they can increase healthcare costs significantly [4]. The increased consumption of antimicrobials and the poor implementation of infection control measures in the hospital setting are the two main causative factors for their emergence. The deep knowledge of the virulent factors that these bacteria produce could provide useful information regarding their spread. Whole-genome sequencing (WGS) has contributed to this aim over recent years [5].

The main problem concerning infections caused by these bacteria is that treatment options are extremely limited, as only a limited number of novel antimicrobials have been launched over the last few years [6]. The molecular epidemiology of infections caused by Gram-negative bacteria is significant as it can determine which of these few new antimicrobial agents could be effective for their treatment [7]. This Special Issue entitled ‘Molecular Characterization of Gram-Negative Bacteria: Antimicrobial Resistance, Virulence and Epidemiology’ sought manuscript submissions that could expand our knowledge of antimicrobial resistance in Gram-negative bacteria. Submissions on the mechanisms of antimicrobial resistance, the presence and function of virulent factors, as well as the molecular epidemiology of Gram-negative bacterial infections were especially encouraged. Ultimately, 12 manuscripts were submitted for consideration for the Special Issue, and they were all accepted for publication. The contributions are briefly analysed below.

In the first contribution, Braspenning et al. examine the frequency and incidence of colistin heteroresistance among a huge number of MDR *K. pneumoniae* strains. The authors have performed WGS and examined the in vitro susceptibility to colistin. Colistin-heteroresistant isolates are shown to have increased sequence type (ST) diversity, while colistin-resistant *K. pneumoniae* isolates are associated with particular STs, such as ST101, ST147 and ST258/ST512.

In the second contribution, Papa et al. attempt to elucidate the differences in virulence features in normoxia and anoxia between MDR and PDR *P. aeruginosa* clinical strains and sensitive strains to antimicrobials, isolated in cystic fibrosis patients. The authors have performed pyocyanin, pyoverdine, protease, zymography and motility assays, as well as biofilm quantification, and they reveal that these features are highly diversified in anaerobiosis, which depicts the condition of cystic fibrosis chronic infection.

In the third contribution, Meletis et al. report the isolation of *bla*_NDM-1_ in three different bacterial species (*K. pneumoniae*, *Proteus mirabilis* and *Morganella morganii*) isolated from a single patient. The authors have used WGS and bioinformatic tools to detect antimicrobial resistance genes and plasmids. They disclose that *bla*_NDM-1_, along with its neighbouring genes, belongs to the same cluster, implying the in vivo transfer of the *bla*_NDM-1_-containing cluster through three different species.

In the fourth contribution, Khalid et al. present the in vitro synergistic effectiveness of 19 different combinations of antimicrobials in 31 New Delhi metallo β-lactamase (NDM) producing MDR isolates of *Enterobacterales*. The authors have used the 2D (two-dimensional) checkerboard method. They have revealed that three combinations of antimicrobials, namely doripenem with cefoxitin, doripenem with streptomycin, and imipenem with cefoxitin, are effective against these MDR isolates, suggesting further in vivo and pharmaceutical studies need to be conducted.

In the fifth contribution, Zarras et al. study 24 carbapenem-resistant *K. pneumoniae* strains, isolated from a single healthcare institution. The authors have applied WGS and various pieces of bioinformatics software to identify antimicrobial resistance genes, plasmids and STs. In addition, they have compared multiple genome alignments and identify core genome single-nucleotide polymorphism sites (SNPs). The authors disclose that the isolates are assigned to seven different phylogenetic branches, with the phylogeny changing every seven SNPs.

In the sixth contribution, Zhang et al. determine the prevalence and molecular characteristics of *bla*_CTX-M-55_-positive *Escherichia coli* isolated from duck–fish polyculture farms in Guangzhou, China. The authors have used WGS, southern hybridization and pulse field gel electrophoresis (PFGE) to detect the possible horizontal transfer and clonal dissemination of *bla*_CTX-M-55_. The authors have revealed that the F18:A-:B1 plasmid might have major significance in the transmission of *bla*_CTX-M-55_ in *E. coli*.

In the seventh contribution, Gupta et al. report the cloning and analysis of *phI*D (involved in the biosynthesis of non-volatile metabolite phloroglucinol) from soil-borne Gram-negative bacteria *Pseudomonas* spp. In addition, the authors have analysed the structural details of the PHLD protein, providing novel strategies for the combinatorial biosynthesis of natural but pharmaceutically important metabolites with enhanced antibacterial and biocontrol effects.

In the eighth contribution, Osman et al. present the molecular analysis of 86 *K. pneumoniae* strains, with most of them being MDR, isolated from different hospitals of Khartoum, Sudan. The authors have performed WGS to detect virulence and resistome profiles. *Ybt9* is the most common virulence gene detected, while the authors report the detection of various antimicrobial resistance genes. In addition, transmissions between patients are found to be rare.

In the ninth contribution, Hao et al. present the differences in the molecular characteristics and the proteomes of sensitive, MDR and XDR *K. pneumoniae* strains. The authors’ enrichment analysis has revealed that a majority of differentially expressed proteins are involved in various metabolic pathways which are beneficial to the evolution of antimicrobial resistance in *K. pneumoniae*.

In the tenth contribution, Karruli et al. present a literature review to assess the antimicrobial activity of cefiderocol and sulbactam-durlobactam against carbapenem-resistant *A. baumannii* (CRAB). The authors have concluded that cefiderocol is non-inferior to colistin/other treatments for CRAB infections and displays a better safety profile. Combination treatment is not correlated with improved outcomes compared to monotherapy. Sulbactam-durlobactam could also be another valuable option against CRAB infections.

In the eleventh contribution, Karampatakis et al. review the dynamic evolution of the molecular epidemiology of carbapenem-resistant *K. pneumoniae* (CRKP), its virulence factors and the latest updates for the treatment of CRKP infections. In addition, they report the latest guidelines for treating these infections, as proposed by international organisations.

In the twelfth contribution, Li et al. describe the genetic and structural compositions of the five secretion systems that exist in *A. baumannii*. The authors have underlined the importance of these systems in the fitness and pathogenesis of *A. baumannii*, and their contribution to the emergence of antimicrobial resistance.

In conclusion, the manuscripts published in this Special Issue reveal recent data which contribute to the better understanding of the virulence of MDR Gram-negative bacteria, and they provide important updates on the evolution of their molecular epidemiology at a global level.

## List of Contributions

1. Braspenning, A.J.M.M.; Rajakani, S.G.; Sey, A.; El Bounja, M.; Lammens, C.; Glupczynski, Y.; Malhotra-Kumar, S. Assessment of Colistin Heteroresistance among Multidrug-Resistant Klebsiella pneumoniae Isolated from Intensive Care Patients in Europe. *Antibiotics* **2024**, *13*, 281. https://doi.org/10.3390/antibiotics13030281.
2. Papa, R.; Imperlini, E.; Trecca, M.; Paris, I.; Vrenna, G.; Artini, M.; Selan, L. Virulence of *Pseudomonas aeruginosa* in Cystic Fibrosis: Relationships between Normoxia and Anoxia Lifestyle. *Antibiotics* **2023**, *13*, 1. https://doi.org/10.3390/antibiotics13010001.
3. Meletis, G.; Malousi, A.; Tychala, A.; Kassomenaki, A.; Vlachodimou, N.; Mantzana, P.; Metallidis, S.; Skoura, L.; Protonotariou, E. Probable Three-Species In Vivo Transfer of *bla(NDM-1)* in a Single Patient in Greece: Occurrence of NDM-1-Producing *Klebsiella pneumoniae, Proteus mirabilis*, and *Morganella morganii*. *Antibiotics* **2023**, *12*, 1206. https://doi.org/10.3390/antibiotics12071206.
4. Khalid, S.; Migliaccio, A.; Zarrilli, R.; Khan, A.U. Efficacy of Novel Combinations of Antibiotics against Multidrug-Resistant-New Delhi Metallo-Beta-Lactamase-Producing Strains of Enterobacterales. *Antibiotics* **2023**, *12*, 1134. https://doi.org/10.3390/antibiotics12071134.
5. Zarras, C.; Karampatakis, T.; Pappa, S.; Iosifidis, E.; Vagdatli, E.; Roilides, E.; Papa, A. Genetic Characterization of Carbapenem-Resistant *Klebsiella pneumoniae* Clinical Isolates in a Tertiary Hospital in Greece, 2018–2022. *Antibiotics* **2023**, *12*, 976. https://doi.org/10.3390/antibiotics12060976.
6. Zhang, L.J.; Yang, J.T.; Chen, H.X.; Liu, W.Z.; Ding, Y.L.; Chen, R.A.; Zhang, R.M.; Jiang, H.-X. F18:A-:B1 Plasmids Carrying bla(CTX-M-55) Are Prevalent among *Escherichia coli* Isolated from Duck-Fish Polyculture Farms. *Antibiotics* **2023**, *12*, 961. https://doi.org/10.3390/antibiotics12060961.
7. Gupta, P.; Dash, P.K.; Sanjay, T.D.; Pradhan, S.K.; Sreevathsa, R.; Rai, R. Cloning and Molecular Characterization of the *phlD* Gene Involved in the Biosynthesis of “Phloroglucinol”, a Compound with Antibiotic Properties from Plant Growth Promoting *Bacteria Pseudomonas* spp. *Antibiotics* **2023**, *12*, 260. https://doi.org/10.3390/antibiotics12020260.
8. Osman, E.A.; Yokoyama, M.; Altayb, H.N.; Cantillon, D.; Wille, J.; Seifert, H.; Higgins, P.G.; Al-Hassan, L. Klebsiella pneumonia in Sudan: Multidrug Resistance, Polyclonal Dissemination, and Virulence. *Antibiotics* **2023**, *12*, 233. https://doi.org/10.3390/antibiotics12020233.
9. Hao, L.; Yang, X.; Chen, H.; Mo, Z.; Li, Y.; Wei, S.; Zhao, Z. Molecular Characteristics and Quantitative Proteomic Analysis of Klebsiella pneumoniae Strains with Carbapenem and Colistin Resistance. *Antibiotics* **2022**, *11*, 1341. https://doi.org/10.3390/antibiotics11101341.
10. Karruli, A.; Migliaccio, A.; Pournaras, S.; Durante-Mangoni, E.; Zarrilli, R. Cefiderocol and Sulbactam-Durlobactam against Carbapenem-Resistant *Acinetobacter baumannii*. *Antibiotics* **2023**, *12*, 1729. https://doi.org/10.3390/antibiotics12121729.
11. Karampatakis, T.; Tsergouli, K.; Behzadi, P. Carbapenem-Resistant *Klebsiella pneumoniae*: Virulence Factors, Molecular Epidemiology and Latest Updates in Treatment Options. *Antibiotics* **2023**, *12*, 234. https://doi.org/10.3390/antibiotics12020234.
12. Li, P.; Zhang, S.; Wang, J.; Al-Shamiri, M.M.; Han, B.; Chen, Y.; Han, S.; Han, L. Uncovering the Secretion Systems of *Acinetobacter baumannii*: Structures and Functions in Pathogenicity and Antibiotic Resistance. *Antibiotics* **2023**, *12*, 195. https://doi.org/10.3390/antibiotics12020195.
